# Supplementary material for: Intrinsic Water Transport in Moisture-Capturing Hydrogels
Source: Nano Lett. 2024 Mar 4;24(13):3858–65. doi: 10.1021/acs.nanolett.3c04191 (PMC10996070; doi:10.1021/acs.nanolett.3c04191)
Supplement: Supplementary file 1 — nl3c04191_si_001.pdf [file nl3c04191_si_001.pdf]

Supporting Information:

Intrinsic water transport in moisture-capturing hydrogels

Gustav Graeber[1,2,,#], Carlos D. Díaz-Marín[1,#], Leon C. Gaugler[1] and Bachir El Fil[1]*

[1]Device Research Laboratory, Department of Mechanical Engineering, Massachusetts Institute of Technology, Cambridge, Massachusetts 02139, United States

[2]Graeber Lab for Energy Research, Department of Chemistry, Humboldt-Universität zu Berlin, 12489 Berlin, Germany

[#]Equal contribution

*gustav.graeber@hu-berlin.de

1. Hydrogel synthesis

We synthesized hydrogels using a one-pot synthesis approach at room temperature. Specifically, we prepared hydrogel samples with different amounts of lithium chloride (LiCl, anhydrous, purity > 99%). Namely, we synthesized gels with 0, 1, and 5 g of LiCl per g of acrylamide (AM), which we denote as $g_{LiCl}\ g_{AM}^{-1}$. We started a typical synthesis with 100 g of deionized water (DIW) in which we dissolved 0 g, 8.36 g or 41.8 g of lithium chloride (LiCl, anhydrous, purity > 99%) to obtain the three different hydrogels studied here, namely, the 0 $g_{LiCl}\ g_{AM}^{-1}$, 1 $g_{LiCl}\ g_{AM}^{-1}$, and the 5 $g_{LiCl}\ g_{AM}^{-1}$ samples, respectively. After mixing the salt into the water using a magnetic stirrer, we allowed the mixture cool back down to room temperature in a closed beaker. Subsequently, we added 8.36 g AM monomer and mixed the solution for around ten minutes until the monomer was fully dissolved. AM is a hazardous material which should be handled with care in a fume hood. While continuously stirring, we added 5 mg *N,N'*-methylenebisacrylamide (MBA) as crosslinker and 14.2 mg ammonium persulfate (APS) as initiator. Finally, as accelerator, we added 12 µL *N*,*N*,*N'*,*N'*-tetramethylethylendiamin (TEMED). We quickly filled the pre-gel solution into glass petri dishes (VWR, inner diameter 89 mm), closed the dish with a glass lid and sealed it with parafilm. We let the gels cure at room temperature for 24 hours. The as-prepared hydrogel had a thickness of 9 mm. Prior to adding the pregel, we weighed the bottom of the glass petri dishes in order to subtract the weight of the glass at a later stage during the analysis.

We note that, as highlighted in previous works,[1,2] increasing the salt content beyond 5 g of LiCl per g of monomer will lead to higher uptakes. In this work, we have used 5 g LiCl per g of AM as the highest salt content as it reliably allowed us to synthesize hydrogels in a manner compatible with our molding process. For higher salt loading, gelation occurred too quickly once the accelerator was added, preventing us from pouring the pre-gel solution into the molds before gelation.

2. Environmental chamber

The sorption and desorption experiments were performed in a vacuum chamber, see **Figure S1**. The vacuum chamber is constructed from stainless steel with a volume of around one cubic foot and features a variety of ports to add sensors and different lines to add vapor or pull vacuum. A rotary vacuum pump (2010 SD, Adixen) with an ultimate pressure of 0.2 Pa is connected to the chamber via a cold-trap. The pump can be isolated from the chamber using a valve. To generate a pure-vapor environment, a boiler is linked to the chamber via a vapor line. In order to isolate the boiler from the chamber, we installed a ball valve (Swagelok) that was equipped with a quarter turn actuator (MDM-M-060DT5-1-SS-42GVC, Hanbay Inc.) for automatic opening and closing of the valve. For more precise control of vapor inflow, a high flow metering valve (Swagelok) was added in between the ball valve and the chamber. Both the vacuum chamber and the boiler can be temperature controlled through a PID controller. We attached a Pirani gauge (925 Micro Pirani, MKS) to measure pressures from atmosphere down to 10^{-5} torr. Using a proprietary software from MKS, we were able to log the Pirani gauge measurements. In extension to the 925 Micro Pirani, we added a manual bellows sealed valve (Kurt J. Lesker) to be able to vent the chamber to atmospheric pressures after an experiment. In order to track vapor pressures during an experiment, we installed a Baratron capacitance manometer (E27F, 100 torr pressure range, MKS) with an accuracy of 0.12%. A capacitance manometer is insensitive to the gas composition and hence works in both air and vapor environments. The Baratron outputs a voltage signal (0-10 VDC) that is directly proportional to pressure. We transmitted this signal into a digital readout controller (PDR 2000, MKS). During adsorption, we wanted to create an environment with a vapor pressure of 2970 Pa, which is equivalent to 70% relative pressure at 30 °C. Whenever the vapor pressure fell below 2940 Pa, the pressure controller sent a signal to a quarter turn actuator, which then opened the valve between the boiler and the environmental chamber to let more vapor enter the chamber. Whenever the vapor pressure exceeded 3000 Pa, the controller closed the valve again. Thereby, the vapor pressure in the chamber remained between 69% and 71% throughout the experiment. To log the pressure data, we used a DAQ (NI-9425, National Instruments) and LabVIEW code that reads the voltage signal and translates it into the corresponding pressure. On the inside of the chamber, we built a rack with three levels. The shelves were made from 316 stainless steel sheets (2 mm thickness, McMaster-Carr). The shelves were separated from each other using vacuum-compatible optical posts with a length of 4.5 in (Thorlabs). The top-shelve functions as a roof to protect the sample on the second shelve from direct exposure to vapor and condensate entering the chamber through the vapor-line. On each of the two other shelves, we placed scales with precision of 0.01 g and an RS-232C output (EK-610i Compact Balance, A&D Engineering) to monitor the weight of two hydrogel samples as a function of the environmental conditions. The data that is collected from the two scales is transmitted via a USB feedthrough to a laptop computer outside the vacuum chamber. We used proprietary software provided by A&D Engineering to log the data of both scales simultaneously. To control and track temperature in the chamber, we installed a type K thermocouple feedthrough (Kurt J. Lesker). One thermocouple

each measures the temperature above the samples, whereas a third provides feedback to the PID controller (CN7800, Omega). The PID controller is connected to a solid-state relay (SSRL240DC25, Omega) that transmits current to heating pads mounted to the outside of the vacuum chamber.

We measured the leak rate of the chamber with pressure sensors as well as the other feedthroughs installed to check for any leaks. The leak rate was less than 1 Pa h^{-1}. We also measured the leak rate combined with the out-gassing rate after the rack, scales, and required cables were installed in the chamber. After pumping-down the chamber for several days, we achieved a combined leak and out-gassing rate of 4.5 Pa h^{-1} at pressures as low as 2.3 mtorr. In order to create a pure-vapor environment, we set-up a boiler that is connected to the vacuum chamber via a vapor line. The boiler is constructed from stainless steel and has a capacity of about 4 L. We attached two vapor outlets using Swagelok tubing. One of the vapor outlets is connected to the chamber, whereas the other is used for de-gassing the boiler prior to operation. The vapor line that leads from the boiler to the chamber is heated with a heating coil to avoid condensation during vapor delivery. Further, we attached a direct line from the vacuum pump to the vapor line in order to evacuate any remaining air from the boiler before an experiment. The second vapor line includes a diaphragm sealed valve (Swagelok) used for de-gassing and a proportional relief valve (Swagelok). If the pressure in the boiler rises above 2 bar, the safety valve opens and releases steam. In addition to the two vapor outlets, we installed a water inlet. The tube of the water inlet reaches down to the bottom of the boiler. To fill the boiler, we used a peristaltic pump (Easy-Load II, Masterflex) and deionized water (ACS Reagent Grade, VWR). The temperature of the boiler is regulated through a PID controller. Two J-type thermocouples reach down into the boiler to measure the temperature of the water, of which one is looped back to the PID. To ensure that no air enters the chamber through the boiler, we had to ensure that the boiler is entirely leak-tight. We put the boiler under 2 bar overpressure by connecting it to a helium tank. Subsequently, we used a helium leak detector (ASM 142, Pfeiffer Adixen) in sniffing mode to check that no helium escapes from the boiler or any of the adjacent vapor lines or water lines.

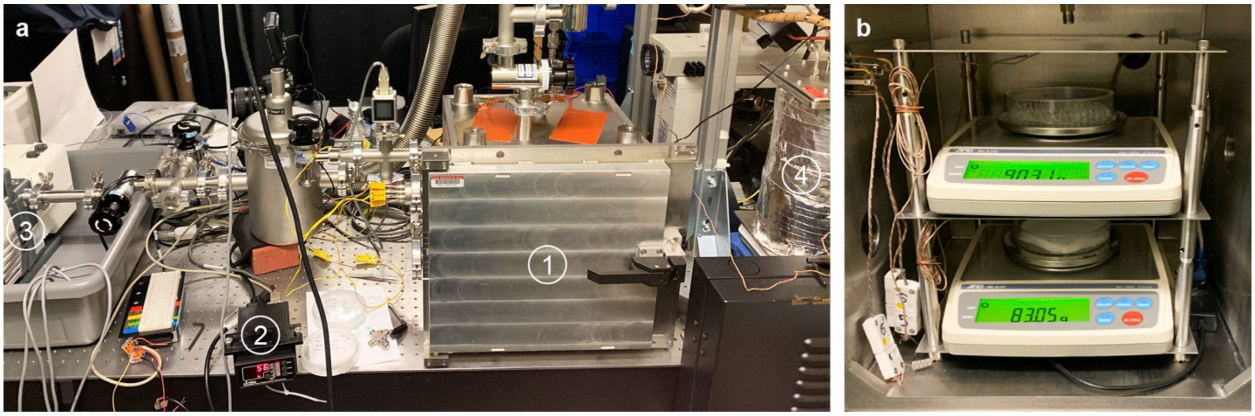

Figure S1. Characterization of lithium-chloride-loaded polyacrylamide hydrogels. a, Experimental setup to study sorption kinetics in a pure-vapor environment consisting of a vacuum chamber (1), a pressure sensor (2), a vacuum pump (3) and a boiler (4). **b**, Inside-view of the environmental chamber featuring two scales to simultaneously characterize sorption of two hydrogel samples.

3. Crystallization

As water desorbed from the hydrogels, the salt concentration increases. Once the concentration of the salt solution reaches its solubility limit in the hydrogel, the salt crystallizes into solid hydrates. This is a spatially varying process as the crystallization occurs first in the parts with lower water concentrations. Here, we estimate the uptake corresponding to the crystallization condition assuming a uniform crystallization.

We first define the uptake $U(t)$ as

$$U(t) = \frac{m_{\mathrm{w}}(t)}{m_{\mathrm{poly}} + m_{\mathrm{s}}} \tag{S1}$$

where m_{w}, m_{poly}, and m_{s} are the masses of water, polymer, and salt in the hydrogel at a given time. Equation (S1) can be rewritten as

$$U(t) = \frac{m_{\mathrm{w}}(t)/m_{\mathrm{s}}}{m_{\mathrm{poly}}/m_{\mathrm{s}} + 1} \tag{S2}$$

where $m_{\mathrm{poly}}/m_{\mathrm{s}}$ is a constant which we control during synthesis by selecting the salt to monomer ratio.

At crystallization, the water to salt mass fraction $m_{\mathrm{w}}(t)/m_{\mathrm{s}}$ reaches the solubility limit. For lithium chloride, we estimate this value as 1.183.[3] Therefore, for a hydrogel with 5 $g_{LiCl}\ g_{AM}^{-1}$ with $\frac{m_{\mathrm{poly}}}{m_{\mathrm{s}}} = 0.2$, we estimate the crystallization uptake as ≈1 $g_{H2O}\ g_{dry}^{-1}$, as considered in the manuscript.

4. Derivation of desorption and absorption uptake

Here, we derive expressions for the desorption and absorption uptakes, $U_{\mathrm{des}}(t)$ and $U_{\mathrm{abs}}(t)$, respectively. We focus on mass transport once the salt crystals have deliquesced into a solution. This allows us to consider the one-dimensional diffusion of liquid water in the hydrogel. We note that the hydrogel is exposed to vapor only from the top. Therefore, the flux of water from any other side of the hydrogel is considered zero. Additionally, the concentration of liquid at the top of the hydrogel is considered constant throughout the entire experiment. This is a consequence of the negligible resistance to vapor transport outside of the hydrogel due to the pure-vapor environment the experiments are conducted in. With these assumptions, the solution to the diffusion equation for the water concentration in the hydrogel $C(x,t)$ is[4,5]

$$\frac{C - C_\infty}{C_0 - C_\infty} = \frac{2}{\pi}\sum_{n=0}^{\infty} \frac{(-1)^n}{n+\frac{1}{2}} \exp\left(-\frac{\left(n+\frac{1}{2}\right)^2 \pi^2 D t}{L^2}\right) \cos\left(\left(n+\frac{1}{2}\right)\frac{\pi x}{L}\right) \tag{S3}$$

where C_∞ and C_0 are the concentration at the top and initial concentration, respectively. D is the diffusivity of water in the hydrogel and L is the thickness of the hydrogel. x is the spatial coordinate along the hydrogel thickness as measured starting from the bottom of the hydrogel. In writing Equation (S3), we assumed that the diffusivity and the thickness are constant.

We can calculate the uptake as

$$\begin{aligned} U(t) &= \frac{A}{(m_{\mathrm{poly}} + m_{\mathrm{s}})}\int_0^L C dx \\ &= U_\infty + \frac{2(U_0 - U_\infty)}{\pi^2}\sum_{n=0}^{\infty} \frac{1}{\left(n+\frac{1}{2}\right)^2} \exp\left(-\frac{\left(n+\frac{1}{2}\right)^2 \pi^2 D t}{L^2}\right) \end{aligned} \tag{S4}$$

where A is the hydrogel cross-section area, $U_\infty = C_\infty AL/(m_{\mathrm{poly}} + m_{\mathrm{s}})$ is the uptake at the top of the hydrogel, and $U_0 = C_0 AL/(m_{\mathrm{poly}} + m_{\mathrm{s}})$ is the initial uptake.

For desorption, $U_\infty = 0$ and we obtain

$$U_{\mathrm{des}}(t) = +\frac{2U_0}{\pi^2}\sum_{n=0}^{\infty} \frac{1}{\left(n+\frac{1}{2}\right)^2} \exp\left(-\frac{\left(n+\frac{1}{2}\right)^2 \pi^2 D_{\mathrm{des}} t}{L^2}\right) \tag{S5}$$

as used in Equation (1) of the main text.

For absorption, we model the process starting from the crystallization condition ($U_0 = U_{\mathrm{cryst}}$) and obtain

$$U_{\mathrm{abs}}(t) = U_\infty + \frac{2(U_0 - U_\infty)}{\pi^2}\sum_{n=0}^{\infty}\frac{1}{\left(n+\frac{1}{2}\right)^2}\exp\left(-\frac{\left(n+\frac{1}{2}\right)^2\pi^2 D_{\mathrm{abs}} t}{L^2}\right) \tag{S6}$$

as used in Equation (2) in the main text.

During absorption, the boundary uptake U_∞ can be computed from the equilibrium of lithium chloride solutions as a function of relative pressure. From Equation (S2) evaluated at equilibrium conditions

$$U_\infty = \frac{(1-\xi_\infty)/\xi_\infty}{m_{\mathrm{poly}}/m_{\mathrm{s}} + 1} \tag{S7}$$

where ξ_∞ is the salt mass ratio in the solution in equilibrium conditions at a partial pressure of 0.7, which is the experimental condition studied in this work. We estimate this value using the empirical fit by Conde for lithium chloride solutions to be 0.1956.[6] With this, we estimate $U_\infty = 3.427$ g_{H2O} g_{dry}^{-1}.

Beyond the deliquescence limit, i.e., when the salts crystallize, mass transport will no longer be described by Equations (S5) and (S6). For salt in hydrogels, previous works have hypothesized that different mechanisms such as the adsorption of additives into the crystals, stochastic nucleation, and hindered diffusion of ions, can dramatically change the crystallization kinetics and the morphology of the crystals.[7] Furthermore, previous experimental studies have observed changes of the salt crystals size as salts are cycled through absorption and desorption.[8] This adds additional complexity into mathematically describing the mass transport after crystallization.

5. Average water absorption rate

We estimate the average absorption rate $\dot{M}$ by dividing the mass change as the system equilibrates from its initial to its final condition by the characteristic timescale of this process. This timescale is (L^2/D_{abs}) as the process is diffusive.

We note that this average absorption rate will be an overestimate, as it takes longer than $t = L^2/D_{\text{abs}}$ to reach steady state. For instance, if we consider $t = L^2/D_{\text{abs}}$ and $U_0 = 0$ in Equation (S6), we obtain

$$U_{\text{abs}}(t = L^2/D_{\text{abs}}) = U_\infty - \frac{2U_\infty}{\pi^2} \sum_{n=0}^{\infty} \frac{\exp\left(-\left(n + \frac{1}{2}\right)^2 \pi^2\right)}{\left(n + \frac{1}{2}\right)^2} = 0.63U_\infty \tag{S8}$$

that is, the uptake reaches 63% of its steady state value in $t = L^2/D_{\text{abs}}$. The uptake will further approach its equilibrium value over higher multiples of L^2/D_{abs}. However, we choose $t = L^2/D_{\text{abs}}$ to describe the absorption rate as it correctly contains the dependency of the timescale with the governing physical properties, namely the diffusivity and the diffusion length scale.

6. Effective diffusion length as a function of porosity and pore radius

Here, we derive an expression for the effective diffusion length as a function of the sample porosity and pore radius for the geometrical design used in our molding process. We consider a unit triangle from our hexagonal patterned pores (**Figure S2**).

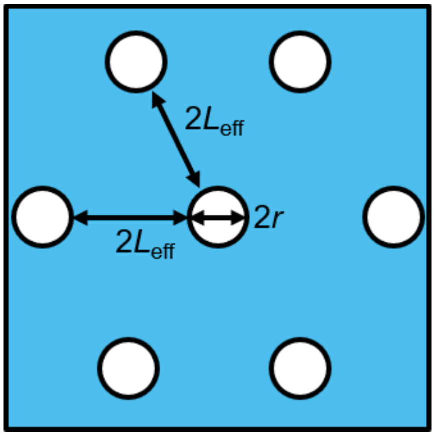

Figure S2. Top view of the pore pattern used in this work. The pores are arranged in a triangular pattern with side $2L_{eff} + 2r$. The pores have a radius r and the sample porosity is ϕ.

This triangle is equilateral with sides $2L_{eff} + 2r$. The triangle area A_t is

$$A_t = \frac{\sqrt{3}}{4}(2L_{eff} + 2r)^2 = \sqrt{3}(L_{eff} + r)^2 \quad \text{(S8)}$$

The area corresponding to pores in this triangle A_p is

$$A_p = \pi r^2 \quad \text{(S9)}$$

The porosity ϕ is then given as

$$\phi = \frac{A_p}{A_t} = \frac{\pi r^2}{\sqrt{3}(L_{eff} + r)^2} \quad \text{(S10)}$$

Solving for L_{eff}

$$L_{eff} = \left(\frac{\pi r^2}{\sqrt{3}\phi}\right)^{0.5} - r, \quad \text{(S11)}$$

For the sample with $\phi = 0.04$ and $r = 425$ µm, $L_{eff} = 2.6$ mm. This effective diffusion distance implies a reduction of the absorption/desorption timescale by 11.98 times, relative to a nonporous hydrogel with a thickness of 9 mm.

7. Fabrication of porous samples

A fully gelated PAM is very tough and cannot be cut easily, not even with a razor blade. In order to create porous LiCl-PAM composites with a rational design, we developed a molding technique that allowed us to shape the gel already during synthesis. We prepared the porous LiCl-loaded PAM hydrogels in three mayor steps. First, we created a mold from acrylic. Second, we used these acrylic molds to cast hairy elastomer templates from polydimethylsiloxane (PDMS). Third, we used these PDMS elastomer templates in glass petri dishes to cast our porous hydrogels.

Step 1: Acrylic molds. To prepare the molds from acrylic, we followed an approach proposed by Alvarado et al..[9] A circular work-piece (diameter of 170 mm) was cut from a sheet of clear cast acrylic of 1/2 inch thickness (McMaster-Carr) using a 120 W laser-cutter (Fusion M2, Epilog Laser). We then used the laser-cutter to cut a triangular array of holes into the acrylic sheet using maximum power and minimum speed during a single exposure. We used spacers to lift the acrylic sheets from the metal bed of the laser cutter to avoid heat related defects on the side of the workpiece that is out-of-focus with the laser-cutter otherwise suffering from the considerable heat accumulation on the metal bed. A mold was constructed by cutting a circular frame and bottom of the same outer dimensions as our workpiece (170 mm) from clear cast acrylic sheets of 1/4 inch and 1/8 inch thickness, respectively. All three components of the acrylic mold were cleaned with ethanol and an air gun. Using acrylic cement (SCIGRIP 16, IPS Corp) the frame was glued to the out-of-focus side of the work-piece, while the bottom was bonded to the in-focus side. By assembling the mold in this orientation, we facilitated the demolding process, which would be impaired by slightly expanding exit holes otherwise. To functionalize the surface of the mold for easier demolding, we employed a silanization process. 200 μL of trichloro(1H,1H,2H,2H-perfluorooctyl)silane (CAS 78560-45-9, Sigma-Aldrich) were mixed with toluene (CAS 108-88-3, Sigma-Aldrich) in a 1:20 volume ratio and vapor deposited on the mold in a seal-tight container at 100 °C for 3 h, thereby substantially reducing the surface energy of the acrylic.

Step 2: Hairy elastomer templates. To prepare the hairy elastomer surfaces, base and curing agent of the PDMS (Sylgard 184, Dow Corning) were mixed in a 10:1 weight ratio by stirring (2 min) and the mixture was subsequently de-gassed in a desiccator until all air bubbles had been removed from the PDMS (20 min). After the functionalized acrylic mold had cooled down to room temperature, the PDMS was poured into the mold. To remove all the trapped air and allow PDMS to penetrate the holes of the array, the PDMS was again de-gassed thoroughly (1 h). The PDMS was then cured overnight at 80 °C. Before the release of the PDMS, the mold was soaked in pure ethanol. Ethanol wets the PDMS surface and acts as a lubrication layer. To avoid fractures of the hairs or base, the PDMS needs to be demolded without excessive force. We used a glass petri dish (10 cm outer diameter, VWR) to assemble a PDMS mold for the PAM synthesis. The bottom part of the glass petri dish has an inner diameter of 89 mm. To cut away the excessive part of the PDMS, we employed a hammer-driven hole punch with a diameter of 3.5 in (88.9 mm) suitable for soft materials (McMaster-Carr). **Figure S3** shows the final mold used to prepare the porous LiCl-

loaded PAM hydrogels. This mold consists of the glass petri dish to hold the gel and the PDMS hairy surface to create the vertical pores.

Step 3: Porous hydrogels. To prepare the porous hydrogels, we followed the same recipe as for the non-porous samples. We quickly filled the pre-gel solution into glass petri dishes that contained the hairy elastomer templates, closed the dish with a glass lid and sealed it with parafilm. We let the gels cure at room temperature for 24 hours. The as-prepared hydrogel had a thickness of 9 mm. Once the hydrogels were fully cured, we carefully removed the hairy elastomer templates and placed the porous sample into a glass petri dish. Prior to transferring the porous hydrogel, we weighed the bottom of the glass petri dishes in order to subtract the weight of the glass at a later stage during the analysis.

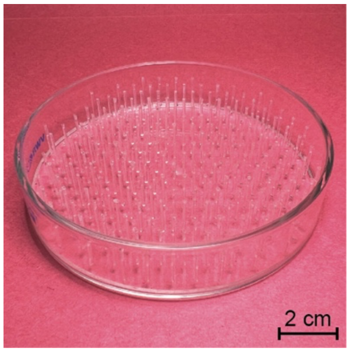

Figure S3. Final mold used to prepare the porous LiCl-loaded PAM hydrogels. It features pillars (0.85 mm diameter, 12 mm height) in a regular triangular pattern (pitch 4 mm).

References

(1) Graeber, G.; Díaz-Marín, C. D.; Gaugler, L. C.; Zhong, Y.; Fil, B. El; Liu, X.; Wang, E. N. Extreme Water Uptake of Hygroscopic Hydrogels through Maximized Swelling-Induced Salt Loading. *Advanced Materials* **2023**, 2211783.

(2) Díaz-Marín, C. D.; Zhang, L.; Lu, Z.; Alshrah, M.; Grossman, J. C.; Wang, E. N. Kinetics of Sorption in Hygroscopic Hydrogels. *Nano Lett* **2022**, *22* (3), 1100–1107.

(3) Lithium Chloride | LiCl | CID 433294 - PubChem https://pubchem.ncbi.nlm.nih.gov/compound/Lithium-Chloride (accessed Oct 22, 2023).

(4) Mills, A. F. *Heat and Mass Transfer*; McGraw-Hill: New York, N. Y., 1994.

(5) Park, H.; Haechler, I.; Schnoering, G.; Ponte, M. D.; Schutzius, T. M.; Poulikakos, D. Enhanced Atmospheric Water Harvesting with Sunlight-Activated Sorption Ratcheting. *ACS Appl Mater Interfaces* **2022**, *14* (1), 2237–2245.

(6) Conde, M. R. Properties of Aqueous Solutions of Lithium and Calcium Chlorides: Formulations for Use in Air Conditioning Equipment Design. *International Journal of Thermal Sciences* **2004**, *43* (4), 367–382.

(7) Schroeder, T. B. H.; Aizenberg, J. Patterned Crystal Growth and Heat Wave Generation in Hydrogels. *Nature Communications 2022 13:1* **2022**, *13* (1), 1–8.

(8) Martin, A.; Lilley, D.; Prasher, R.; Kaur, S. Particle Size Optimization of Thermochemical Salt Hydrates for High Energy Density Thermal Storage. *Energy & Environmental Materials* **2023**, e12544.

(9) Alvarado, J.; Comtet, J.; De Langre, E.; Hosoi, A. E. Nonlinear Flow Response of Soft Hair Beds. *Nature Physics 2017 13:10* **2017**, *13* (10), 1014–1019.
